# Is stemflow a vector for the transport of small metazoans from tree surfaces down to soil?

**DOI:** 10.1186/s12898-018-0198-4

**Published:** 2018-10-11

**Authors:** Christoph Ptatscheck, Patrick Connor Milne, Walter Traunspurger

**Affiliations:** Animal Ecology, Bielefeld University, Konsequenz 45, 33615 Bielefeld, Germany

**Keywords:** Forest ecosystems, Forest soil, Canopy, Nematodes, Rotifers, Collembolans

## Abstract

**Background:**

Stemflow is an essential hydrologic process shaping the soil of forests by providing a concentrated input of rainwater and solutions. However, the transport of metazoans by stemflow has yet to be investigated. This 8-week study documented the organisms (< 2 mm) present in the stemflow of different tree species. Because the texture of the tree bark is a crucial determination of stemflow, trees with smooth bark (*Carpinus betulus* and *Fagus sylvatica*) and rough bark (*Quercus robur*) were examined.

**Results:**

Up to 1170 individuals per liter of stemflow were collected. For rotifers and nematodes, a highly positive correlation between abundance and stemflow yield was determined. Both taxa were predominant (rotifers: up to 70%, nematodes: up to 13.5%) in the stemflow of smooth-barked trees whereas in that of the oak trees collembolans were the most abundant organisms (77.3%). The mean number of organisms collected per liter of stemflow from the two species of smooth-barked trees was very similar. A higher number of nematode species was found in the stemflow of these trees than in the stemflow of rough-barked oak and all were typical colonizers of soil- and bark-associated habitats.

**Conclusion:**

This pilot study showed for the first time that stemflow is a transport vector for numerous small metazoans. By connecting tree habitats (e.g., bark, moss, lichens or water-filled tree holes) with soil, stemflow may influence the composition of soil fauna by mediating intensive organismal dispersal.

## Background

Stemflow is the proportion of rain water that is held back by leaves or branches and drained by the stem. By connecting the vegetative canopy with the soil, this hydrologic process has an important impact on the biogeochemical cycles of forest ecosystems [1]. According to Levia and Frost [2], stemflow accounts for approximately 3.5%, 11.3%, and 19.0% of the precipitation in tropical, temperate, and (semi)arid ecoregions, respectively. The quantity and quality of stemflow reflect numerous factors. Thus, in temperate forests, seasonality has a superordinate impact, with more stemflow generated during dormancy than during the growing season. Staelens et al. [3] reported that the minimal amount of precipitation that is required for the generation of stemflow is lower during the growing season than during the dormant season. For example, in *Fagus sylvatica*, 6–16% of the incoming precipitation is funneled during winter and early spring, while only 1–2% runs down the tree during the rest of the year [4]. This difference is explained by the presence during the growing season of leaves, which shield the rain and thereby reduce the stemflow yield, and by the higher amount of precipitation during the dormant season.

In general, the stemflow yield increases with increasing precipitation although during intensive rain events it decreases because water reaches the ground as throughfall [1]. Conversely, when precipitation is low, there may be no stemflow at all because it occurs only when the storage capacity of the tree is exceeded. The texture of the tree bark is a crucial determinant of stemflow. Rough bark has a higher water storage capacity than smooth bark such that a larger amount of precipitation is necessary before the bark is saturated and stemflow is generated [5]. For example, in one study, stemflow along the trunk of rough-barked *Quercus robur* (rough bark) was generated following 5.4 mm of precipitation whereas in smooth-barked *Fagus sylvatica* only 2.8 mm was required [6]. Additional factors influencing the stemflow yield are wind, snowfall, and the morphometry of the tree (e.g., stem diameter and number of branches) [2].

For soil, stemflow provides a concentrated input of rainwater containing solutes and microorganisms. Hence, around the trunk both the amount of moisture and the concentrations of, e.g., Na^+^, K^+^, Ca^++^, and NH4^+^ will be higher [1]. In addition, fungi and bacteria transported by stemflow contribute to shaping the soil microfaunal community [1].

Over the last 30 years, there have been > 900 studies of stemflow, according to the Web of Science (September 2018; search term: stemflow). However, to our knowledge, none of them examined the stemflow-mediated transport of multicellular organisms (metazoans) and the ecological value of this process. The stem surface is colonized by numerous metazoans, with bark, moss, lichens, and water-filled tree holes serving as habitats for rotifers, nematodes, tardigrades, mites, and collembolans [7–10]. Tardigrades, mites, and collembolans can actively disperse along the stem [11–13]. For the reginal transport of small organisms (< 2 mm), the wind is a crucial vector [14]. Especially nematodes can represent > 44% of the aeroplancton and show dispersal rates of > 3000 individuals m^−2^ in 4 weeks [15]. Rough barked trees are important traps for such wind transported organisms [16]. According to this, trees with a pronounced bark texture and covered to varying extents by moss and lichens host a greater number and diversity of arthropods than trees with a smooth bark [8]. Ptatscheck and Traunspurger [17] previously demonstrated that trees can be hotspots for the occurrence of rotifers (mainly bdelloidea) and nematodes, whose densities may reach 500 individual’s cm^−2^. The authors identified several species occupying water-filled tree holes at abundances more than twice as high as those in forest soils [18–20]. Nematodes collected from the tree holes are typical colonizers of soil or connected ecosystems (e.g., moss, bark, and dead wood) and were dominated by bacterial and hyphal feeders [10, 21].

These observations suggested that, by linking stem and soil stemflow may be a vector for the passive dispersal of organisms between these two habitats.

Such dispersal could affect the geneflow, population stability, population dynamic, and thus, the organismal diversity in soil.

Therefore, in this study we examined the stemflow of three species of middle European trees (*Quercus robur, Fagus sylvatica*, and *Carpinus betulus*). Our main goals were: (1) to document the abundances of metazoan taxa transported by stemflow, focusing on nematode diversity, and (2) to document stemflow-mediated transport by different middle European broad-leaved tree species.

As stemflow may be a possible vector for the passive dispersal of organisms we hypothesized that it contains a diverse composition of typical colonizers of bark, moss, lichens, and water-filled tree holes, especially, nematodes and rotifers (mainly bdelloidea) but also of other metazoans (< 2 mm) (hypothesis H1). We also expected that trees differing in the texture of their bark would differ in the abundances and composition of the associated organisms transported by stemflow. Specifically, trees with rough bark should generate less stemflow such that fewer organisms are washed from the stem surface (hypothesis H2.1). Nonetheless, by offering a more structured habitat for organisms, more nematode taxa would be contained in the stemflow of rough-barked (*Q. robur*) than of smooth-barked (*F. sylvatica* and *C. betulus*) trees (hypothesis H2.2). Based on their predominance in water filled tree holes we expected especially bacterial and hyphal feeding nematodes in stemflow (hypothesis H2.3).

## Methods

### Study setup

The investigation was conducted in the vicinity of Bielefeld University for 8 weeks beginning in April 2017. The study site is part of the Teutoburg Forest, Germany (52°02′N, 8°29′E), a lime-beech forest with a mean annual precipitation of 832 mm. Stemflow was collected from trees of three different species (*Fagus sylvatica*, *Carpinus betulus*, and *Quercus robur*). For each species, three trees located next to each other (< 8 m) and whose crowns were in contact were chosen. The basal areas of the stems were 0.07–0.09 m^2^ for *F. sylvatica*, 0.09–0.13 m^2^ for *C. betulus*, and 0.26–0.46 m^2^ for *Q. robur*. The trees were leafless at the beginning of the investigation.

Stemflow collectors were made of hoses (4 cm diameter) from which a section was cut lengthwise. These gutters were wound 1.5 times around the stem, beginning at a height of ~ 1.3 m, and fixed with aquarium silicone. At the lower end of the hose, a smaller, intact hose was installed that drained the stemflow into a covered 10-L plastic bucket.

### Data collection

Depending on the duration of rain events, the buckets were sampled at least once a day, with the volume of the contained stemflow determined using a measuring cylinder. The bucket was thoroughly rinsed with water between samplings. All components were filtered (10 µm), transferred to 250-mL PE bottles, and stored in a fridge at 4 °C for no more than a few days before they were evaluated.

The abundances of nematodes rotifers, tardigrades, mites, and collembolans were determined at 40× magnification using a Leica L2 stereomicroscope. No other arthropod taxa were considered because their input in the stemflow collectors by active movement could not be excluded.

Fifty nematodes per sample were prepared according to Seinhorst [22, 23]. The nematodes were identified to the species level, if possible, based on Leica Dialux microscopy observations (1250× magnification). Feeding types were classified using the methods of Yeates et al. [24] and Traunspurger [25]. For further analysis, the data (stemflow yield and organismal numbers) recorded for 1 week were pooled. Data on the daily amount of rain were obtained from a weather station (Bielefeld-Deppendorf, Germany).

### Data analysis and statistics

To compare the quantity of stemflow from the tree species with different stem diameters, the funneling ratio was calculated according to Herwitz [26], as shown in Eq. (1):

1 $$ F \, = \, V \, / \, \left( {B \, * \, P} \right) $$

where *V* is the stemflow volume, *B* is the basal area of the trunk, and *P* the depth equivalent of incident precipitation. A funneling ratio exceeding 1 indicates that the tree is funneling stemflow from outlying portions of its crown [5].

The Kruskal–Wallis test followed by a Dunn’s test were used to test for differences between the tree species (e.g., funneling ratios and organismal abundances), because a normal distribution (Kolmogorov–Smirnov test) of the data and homogeneity of variances (Levene test) were not applicable in most cases. For the same reason, the Spearman correlation was used to calculate the relationship between stemflow volume and taxon abundance. The figures were created, and the statistical tests performed using SigmaPlot (SystatSofware, version 11).

## Results

### Stemflow funneling

**Fig. 1:**
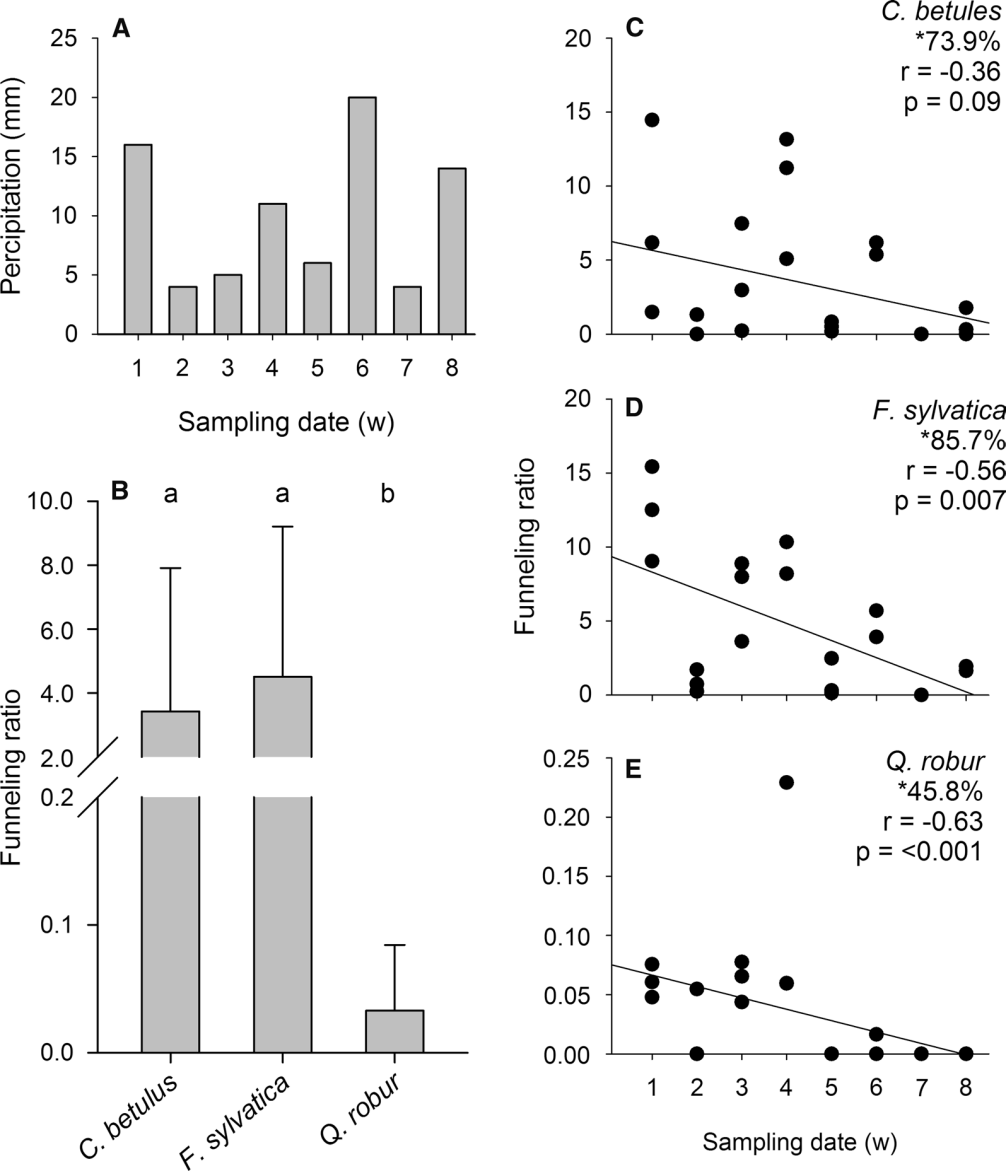
**A** Weekly rain (mm) over duration of study (8 weeks) and **B** mean (± SD) funneling ratio of *Carpinus betulus* (n = 23), *Fagus sylvatica* (n = 21), and *Quercus robur* (n = 24). The different letters above the columns indicate significant differences (Kruskal–Wallis test and post hoc Dunn’s test, p < 0.05). The linear regression (Spearman correlation) between the weekly funneling ratios of *C. betulus* (**C**), *F. sylvatica* (**D**), and *Q. robur* (**E**) and the weekly sampling dates is shown. *Percentage of samplings at which stemflow could be measured after rain events; r = correlation coefficient (Spearman correlation); r = significance level (< 0.05)

During the study, the weekly rain volume was between 4 and 20 mm (Fig. 1A). Due both to storms and to disturbances by deer, during weeks 4, 6, and 8 one tree of *F. sylvatica* and during week 6 one tree of *C. betulus* could not be sampled. Over all, stemflow was collected in 85.7% of the samplings from the stems of *F. sylvatica* and 73.9% and 45.8% of the stems of *C. betulus* and *Q. robur* (Fig. 1C–E). Only during week 7 (4 mm rain) was no stemflow collected from any of the trees.

The maximal stemflow volume collected over the 8-week period from a single *Q. robur* tree was 1 L, and from single trees of *C. betulus* and *F. sylvatica* up to 31.5 and 49.9 L (for weekly stemflow volumes, see Table 1). Stemflow was generated by all *C. betulus* and *F. sylvatica* trees at a threshold of ~ 5 mm of rain. From this amount of rain, the funneling ratios of both tree species were > 1, compared to < 0.23 for *Q. robur* at all sampling dates. The mean funneling ratio of *Q. robur* (0.03 ± 0.05, mean ± SD) was significantly lower than that of *C. betulus* (3.9 ± 4.6) and *F. sylvatica* (5.3 ± 4.8), as shown in Fig. 1B. There were no differences between the funneling ratios and stemflow volumes of *C. betulus* and *F. sylvatica*.

**Table 1:** Organism composition per liter stemflow and stemflow volume from each tree species over 8 weeks

	Number of organisms per liter stemflow over 8 weeks (n = 3, * n = 2)								Percentage	Percentage occurrence
	1	2	3	4	5	6	7	8		
Nematodes										
*C. betulus*	9–19	26	0–10	16–16	5–85	7–15*	0	8–15	10.1	94.1
*F. sylvatica*	11–14	5–21	2–4	17–31*	2–205	0–3*	0	1–2*	13.5	100.0
*Q. robur*	0	0	0	10–40	0	10	0	0	3.8	27.3
Rotifers										
*C. betulus*	11–76	31	6–80	8–31	8–31	73–480*	0	77–400	68.6	100.0
*F. sylvatica*	26–49	28–350	10–30	43–50*	43–50	38–760*	0	10–12*	70.3	100.0
*Q. robur*	3–24	0	0	10–28	10–27	–	0	0	8.5	63.6
Tardigrades										
*C. betulus*	0–6	0	2–40	0–2	0–55	0–1*	0	0–3	6.0	70.6
*F. sylvatica*	1–7	2–50	1–4	3–5*	2–150	0–1*	0	0*	10.0	88.9
*Q. robur*	0–4	0	0	3–8	0	0	0	0	1.1	27.3
Mites										
*C. betulus*	1	0	0–4	0–1	10–80	0–1*	0	5–100	10.0	82.4
*F. sylvatica*	0	0–13	0	0*	8–15	0*	0	0–2*	1.5	100.0
*Q. robur*	0–9	0	10–20	5–40	0	30	0	0	9.2	90.9
Collembolans										
*C. betulus*	0–3	0	1–20	1	20–43	0*	0	5–100	5.4	76.5
*F. sylvatica*	1–2	7–21	1–4	1–2*	10–40	1*	0	1–0*	4.6	72.2
*Q. robur*	24–340	0	110–430	33–46	0	80	0	0	77.3	100.0
Liter stemflow collected										
*C. betulus*	2.2–15.5	0–0.4	0.1–2.5	5.2–10.0	0.1–0.4	10.0*	0.0	0.0–1.8		
*F. sylvatica*	18.5–22.2	0.1–0.6	2.0–5.1	10.0*	0.1–1.3	10.0*	0.0	1.6–1.9*		
*Q. robur*	0.3–0.4	0–0.1	0.1	0.2–0.7	0.0	0.0–0.1	0.0	0.0		

For the collected taxa (nematodes, rotifers, tardigrades, mites, and collembolan), the abundance (range), percentage of the organism’s composition and percentage occurrence are shown for each tree species (*Carpinus betulus*, *Fagus sylvatica*, and *Quercus robur*) (n = 3, * n = 2)

A trend of a decrease in the funneling ratio over the course of the investigation was determined for all three tree species, with a more pronounced reduction for *F. sylvatica* (Fig. 1C–E).

### Organisms transported by stemflow

Overall, more than 10,000 organisms were collected within 8 weeks and all stemflow samples contained organisms. The mean metazoan density calculated from the dataset was (mean ± SD) 153 ± 234 individuals (ind.) L stemflow^−1^ from *C. betulus*, 158 ± 292 ind. L stemflow^−1^ from *F. sylvatica*, and 173 ± 186 ind. L stemflow^−1^ from *Q. robur*. The highest number of transported organisms was from *F. sylvatica* (1170 ind. L stemflow^−1^).

Nematodes, rotifers (bdelloidea), tardigrades, mites, and collembolans were the most common taxa. The taxa most commonly collected from *C. betulus* and *F. sylvatica* were rotifers and nematodes, which were found in nearly all (94.1–100%) samples. Their maximal abundances reached 750 and 205 ind. L stemflow^−1^, representing 68.8% and 10.1% (*C. betulus*) and 70.3% and 13.5% (*F. sylvatica*) of the collected organisms, respectively (Table 1). In *F. sylvatica*, 10% of the organismal composition of the stemflow was made up tardigrades and in *C. betulus* 10% was made up of mites.

**Fig. 2:**
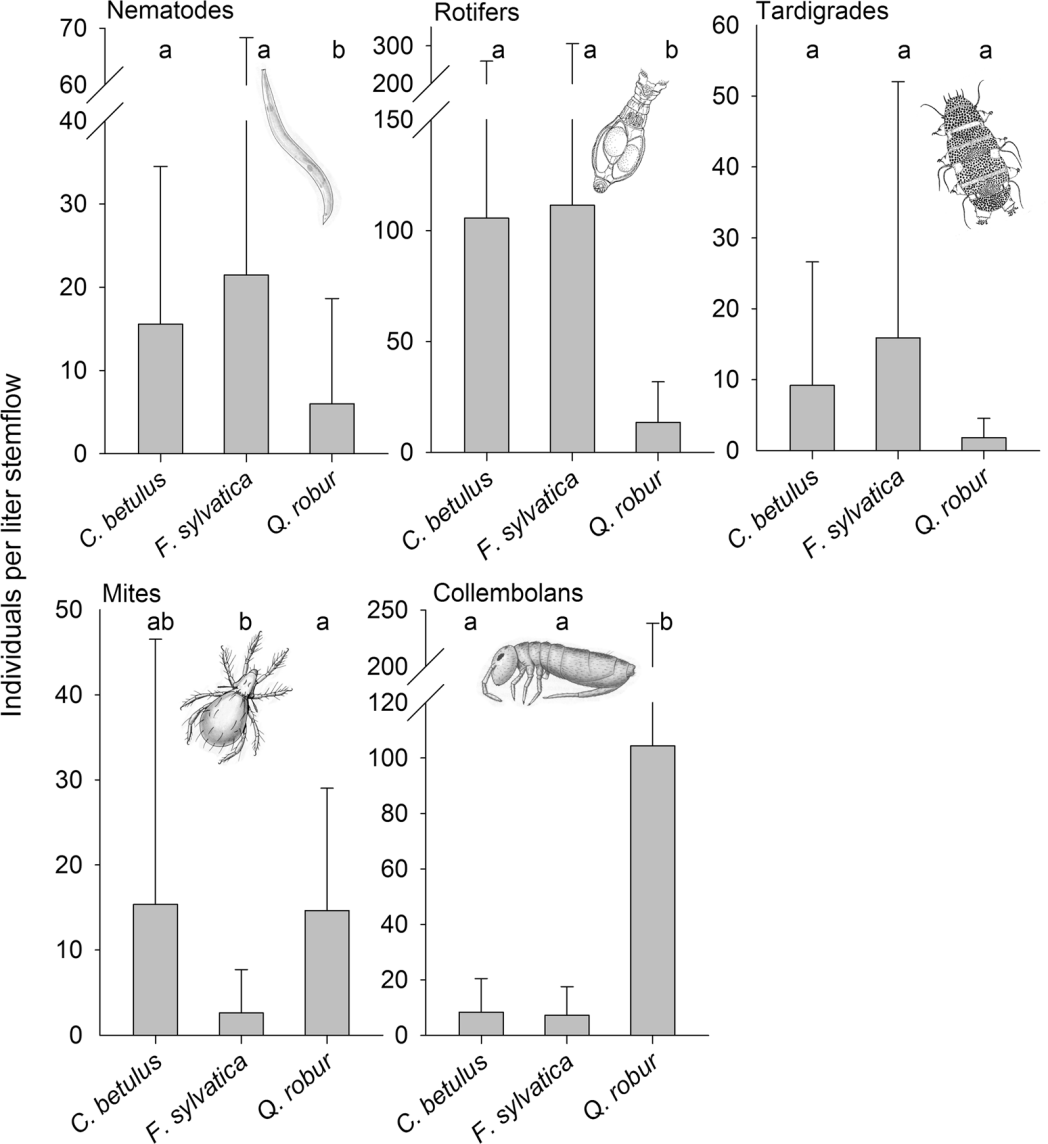
Mean (± SD) number of organisms (nematodes, rotifers, tardigrades, mites, and collembolans) per liter of stemflow of *C. betulus* (n = 17), *F. sylvatica* (n = 18), and *Q. robur* (n = 10). Different letters above the columns indicate significant differences (Kruskal–Wallis test and post hoc Dunn’s test, p < 0.05) (Illustrations of the organisms were modified after McCafferty 1983 [48] and Westerheide et al. 2006 [49])

Nematodes and rotifers were detected less often in the stemflow of *Q. robur* (nematodes: 27.3%; rotifers: 63.7%) than in the stemflows of the other two species and represented only 12.3% of the collected organisms (Table 1). In the stemflows of *C. betulus* and *F. sylvatica*, nematodes reached mean abundances (± SD) of 15.6 ± 19.0 and 21.5 ± 46.9 ind. L stemflow^−1^, and rotifers mean abundances of 105.6 ± 153.9 and 111.4 ± 194.4 ind. L stemflow^−1^. Both taxa were significantly less abundant in the stemflows of the oak trees (Fig. 2), as evidenced by mean abundances (± SD) of nematodes of 6.0 ± 12.7 ind. L stemflow^−1^ and of rotifers of 13.5 ± 18.3 ind. L stemflow^−1^. A similar, but not significant, trend was documented for tardigrades. Mites were most frequent in *F. sylvatica*, but their abundance was lower (1.5% of the total metazoan composition) than in the trees of the other two species (Table 1). By contrast, collembolans predominated (73.3%) in all stemflow samples of Q. *robur* and their mean abundance (122.6 ± 138.3 ind. L stemflow^−1^) was significantly higher than in the stemflows of *C. betulus* and *F. sylvatica* (7.3 ± 10.3 and 8.3 ± 12.1 L stemflow^−1^) (Fig. 2). No significant difference in the abundances of nematodes and rotifers between *C. betulus* and *F. sylvatica* was found for any taxon.

**Fig. 3:**
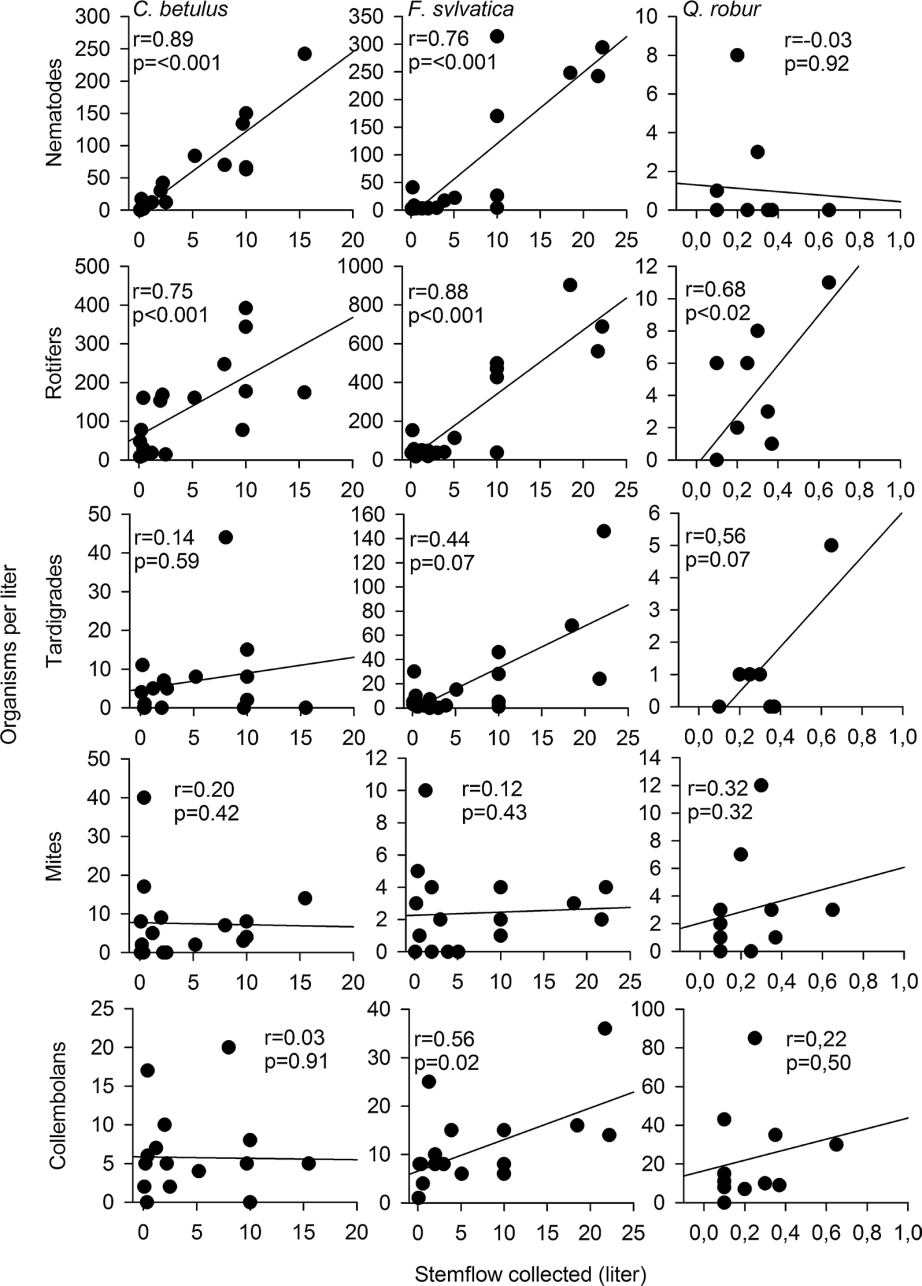
Regression between the volume (L) of stemflow collected from *C. betulus, F. sylvatica,* and *Q. robur* and the abundances (per L) of the different taxa (nematodes, rotifers, tardigrades, mites, and collembolans) in the stemflow. r = correlation coefficient (Spearman correlation); r = significance level (< 0.05)

The number of nematodes and rotifers present in the stemflow samples correlated strongly with the stemflow yield (Fig. 3). This correlation was weaker for other taxa, especially mites and collembolans.

### Nematode composition

**Fig. 4:**
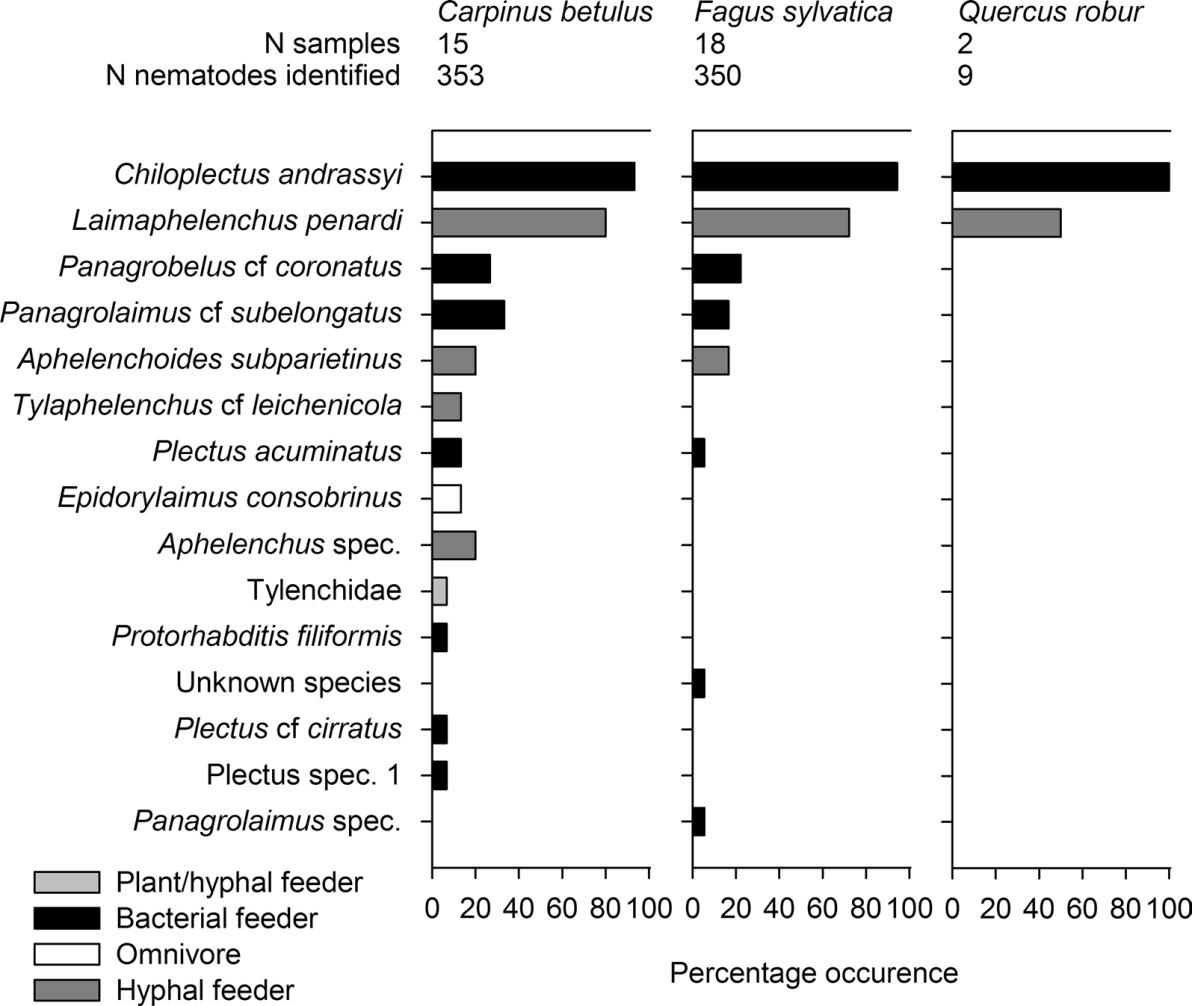
Percentage occurrence of the nematode taxa identified in the stemflows of *C. betulus, F. sylvatica*, and *Q. robur*. The nematodes are listed from the overall most common to the rarest species and according to feeding type

Of the 2357 collected nematodes, 712 could be classified into 15 species (Fig. 4). In the samples from *C. betulus* 13 species and in those from *F. sylvatica* 8 species were detected. However, in those from *Q. robur* only two species from only two samples were present such that a statistical analysis was not possible. For *C. betulus* and *F. sylvatica*, there was no significant difference in the number of nematode species. *Chiloplectus andrassyi* and *Laimaphelenchus penardi* were present in 93.3–100% and 50.0–80.0% of the samples, respectively. Both species clearly dominated the nematode composition of the stemflow, with 46.1% and 30.9% (*C. andrassyi* and *L. penardi*) in *C. betulus*, 63.1% and 30.3% in *F. sylvatica*, and 88.9% and 11.1% in *Q. robur*. Seven nematode species were exclusively found in the stemflow of *C. betulus* and two in the stemflow of *F. sylvatica*. However, these nematode species together made up < 6% of the nematodes collected from both tree species. The majority of nematodes were bacteria-feeding taxa, accounting for 62.6% (*C. betulus*), 68.9% (*F. sylvatica*), and 88.9% (*Q. robur*). Hyphal feeders made up 34.6%, 31.1%, and 11.1%, respectively (Fig. 4).

## Discussion

This is the first known investigation to document the quantity and composition of small organisms transported by the stemflows of three species of Middle European trees. We show that stemflow is a crucial vector for the transport of small metazoans from tree surfaces down to soil.

As demonstrated by Levia et al. [5] and Levia and Herwitz [27], the bark water storage capacity that is affected by the bark texture is an essential factor for the generation of stemflow. According to these findings, in our study *s*ignificantly more stemflow was generated by smooth-barked *C. betulus* and *F. sylvatica* than by rough-barked *Q. robur*. Even at low rain rates, stemflow was successfully measured for the smooth-barked trees. Van Stan et al. [6] similarly determined a lower threshold for the generation of stemflow by *F. sylvatica* (2.8 mm) than by *Q. robur* (5.4) mm and funneling ratios that were ten times higher. In our study, the funneling ratios of the smooth-barked trees were as much as 100 times higher than those of the rough-barked oak. The mean funneling ratios of *C. betulus* and *F. sylvatica* were 3.9 and 5.2, which means an exceeding of the water storage capacity and the contribution of the tree crown to stemflow. For *Q. robur*, the low mean funneling ratio indicated that rainwater was not transferred into stemflow and released mainly as throughfall [1]. According to Van Stan et al. [6], these observations can be explained by the rougher bark texture of *Q. robur*, which allows for a much higher water-storage capacity than is possible by smooth-barked trees such as *F. sylvatica* and, in our study, *C. betulus.*

During the investigation, the funneling ratios of all trees declined as the amount of foliage increase. At the beginning of the study, the trees had only buds but after 8 weeks they were completely foliaged, which reduced the funneling effect [4].

Consistent with hypothesis H1, we identified several taxa that were transported by stemflow (rotifers, nematodes, tardigrades, mites, and collembolans). These organisms, already known from soil systems and tree surfaces, are often associated with adjacent habitats, including moss, lichens, and water-filled tree holes [7, 10, 28–30]. The 15 identified species of nematodes were all colonizers of the soil and trees of forest ecosystems [31–33]. The two predominant nematodes species in our study, *C. andrassyi* and *L. penardi*, were previously shown to be strongly abundant in epiphytic moss from the same sampling site [34] and in water-filled tree holes from other locations [21]. Both species were predominant in aeroplancton collected at the same site [15]. This finding is an important indication how nematodes enter tree habitats. Surprisingly, in water-filled tree holes from the same forest area as the collected stemflow, these two species were not represented [10]. Instead, *Plectus cirratus/acuminatus* dominated, which were, however, rare in stemflow.

The most frequent nematode feeding types of were bacterial feeders and hyphal feeders (H2.3). These feeding types are typical for detritus based water-filled tree holes, with high amount of bacteria and fungi, and for soil [19, 21]. The root-hair feeders and large predacious and omnivorous species that are frequently found in soil and moss were not present in the stemflow. Overall, these results suggest that the individuals collected from the stemflow were indeed flushed from tree components but were able to survive in soil systems.

We collected an exceptionally large number of small juvenile nematodes. For nematodes from *C. betulus* and *F. sylvaticus*, the adult/juvenile ratio was 0.4. By contrast, for nematodes collected from natural beech-forest soil [35] and water-filled tree holes [21] the adult/juvenile ratio was approximately 0.7 and 1, respectively. It may be that juvenile nematodes, because of their small size and weight, are more easily washed away by stemflow, leaving a larger proportion of adults on the stem. However, we found no relationship between the stemflow yield and the age-distribution of the contained nematodes.

Rather, the amount of stemflow had a significant impact on the number of nematodes and rotifers, and a tendency of an impact on tardigrades. With the exception of nematodes from the oak stemflow, more metazoan individuals were found at higher stemflow yield. Moreover, all of these taxa, especially nematodes and rotifers, were collected in significantly larger numbers from the smooth-barked trees. Thus, the textured bark and the lower amount of stemflow of *Q. robur* may better protect the associated organisms from leeching. The same effect has been reported for microbes and arthropods [8, 16]. However, contradicting hypothesis H2.1, the number of collembolans was significantly higher in the stemflow of the oak trees. Furthermore, in total, the number of metazoans per liter stemflow did not differ between the tree species.

The rough bark of *Q. robur* corresponds to a 20% larger surface and a more extensive microstructure than the bark of *F. sylvatica* [8]. Fissured bark supports shading and cooling effects [8] and provides larger amounts of moisture [36]. This microclimate enables the colonization of stems by fungi [16] and moss [37]. This structural and nutritional conditions favor a high diversity as shown for arthropods, including mites and collembolans [8, 30], which may account for the higher abundances of collembolans in the oak stemflow. In addition, most of the bacteria on tree trunks are located in moss [38], which may explain why in this study the majority of the nematode species were bacterial feeders.

Contradicting hypothesis H2.2, relatively few species were present in the stemflow of *Q. robur*. Compared to other tree species (e.g., maple or beech), oak stemflow has a slightly lower pH but contains higher amounts of nitrate, sulfate, and ammonia [39, 40], which promote soil acidification. While the abundances of bacteria-feeding nematodes in forest soil were shown to be negatively affected by the low pH caused by oak stemflow, the effect of acidification on tardigrades and rotifers is either slight or none [41, 42]. A study of water-filled tree holes showed that the pH value could be excluded as a decisive factor influencing the nematode and rotifer communities [10, 17]. Instead, a much larger negative impact was shown for tannins, which are highly concentrated in oak bark and decrease both the movement and survival of nematodes [43]. Nonetheless, an early study reported that the soil of oak forests contained very high nematode densities (> 12 Mio ind. m^−2^) [43].

While our results indicated differences in the composition of organisms transported by stemflow (according to H2), only a thorough investigation of all metazoans in stemflow and on tree surfaces will finally provide insights into the underlying reasons.

Staelens et al. [3] measured an annual stemflow volume of 10,200 L collected from a single *F. sylvatica* (30 m high, 0.68 m breast high diameter, 0.36 m^2^ basal area, 180 m^2^ canopy area). Based on our results, on average 1.6 million metazoans (1.2 million rotifers, 216,000 nematodes, 160,000 tardigrades, 73,000 mites and 25,000 collembolans) are transporter by stemflow per year from a single beech tree. For comparison, mean annual abundances of 650,000 rotifers, 1 million nematodes, 51,000 tardigrades, 31,900 mites and 37,800 collembolans per square meter can be expected in forest soils [20, 28, 44, 45].

Extrapolated to the 1 ha area of our study site and assuming a closed canopy and annual precipitation of 832 mm, 11.7% of which is transferred into stemflow [2], 154 million metazoans are washed down to the soil each year, not including organisms that reach the ground via throughfall. Thus, the input of organisms by stemflow may be a crucial factor for the biodiversity of forest soils that is crucial to ecosystem function [46].

However, this input of tree-living organisms by stemflow is restricted to the soil area around the trunk, as reported by Falkengren-Grerup [47], who demonstrated that an impact of stemflow on soil chemistry is restricted to the 1.5 m around the tree stem.

It should be noted that our study only covered a period of 8 weeks in the spring, when funneling ratios decline. Stemflow yield is higher during the leafless season [4] and increases following strong precipitation events [2]. Thus, a year-long study would better reveal the extent of organismal transport by stemflow. This is especially the case for nematodes and rotifers, whose numbers in this study correlated strongly with the stemflow yield, such that higher densities in stemflow would be expected. Additionally, the combined sampling of organisms from bark, stemflow, and soil would demonstrate the ecological importance of stemflow for soil. These investigations were beyond the scope of our study, but they will provide the basis for more advanced studies.

## Conclusion

Our study provided the first insights into the stemflow-mediated transport of small metazoans. The results confirmed the importance of stemflow in the transport of > 100 tree-living organisms per liter down to the soil. They also suggested that in different forests or even during different seasons the quantity and composition of organisms transported by stemflow vary widely.

Nonetheless, we were able to demonstrate that stemflow is a critical mediator of the distribution of organisms between the canopy, bark, moss, lichens, and water-filled tree holes and the soil. It is therefore an important contributor to gene flow, increased diversity, and the maintenance of ecosystem functions.
